# Metabolic risk and metabolic dysfunction–associated steatotic liver disease and steatohepatitis in cognitive decline: A retrospective cohort study

**DOI:** 10.1371/journal.pone.0353160

**Published:** 2026-07-09

**Authors:** Ahmad Basil Nasir, Yassine Kilani, Mohammad Aldiabat, Omar Abdelghany, Youssef Hafez, Nitin Desai, Mahmoud Y. Madi, Francis G. Wade, Kamran Qureshi, Lewis J. Frey, Adam D. Farmer, Wing-Kin Syn

**Affiliations:** 1 Division of Gastroenterology and Hepatology, Department of Internal Medicine, Saint Louis University School of Medicine, Saint Louis, Missouri, United States of America; 2 Department of Internal Medicine, Saint Louis University School of Medicine, Saint Louis, Missouri, United States of America; 3 Department of Internal Medicine, Washington University, Saint Louis, Missouri, United States of America; 4 Department of Pathology, Saint Louis University School of Medicine, Saint Louis, Missouri, United States of America; 5 Department of Physiology, Faculty of Medicine and Nursing, University of the Basque Country (UPV/EHU), Vizcaya, Spain; Universita degli Studi della Campania Luigi Vanvitelli Scuola di Medicina e Chirurgia, ITALY

## Abstract

**Objective:**

To determine whether metabolic risk factors (MRFs), metabolic dysfunction–associated steatotic liver disease (MASLD) and metabolic dysfunction–associated steatohepatitis (MASH) are associated with incident mild cognitive impairment (MCI), vascular dementia (VD), and Alzheimer disease (AD).

**Design:**

Retrospective cohort study using TriNetX (2003–2023) with Propensity score matching.

**Setting:**

Multicenter, population-based sample from 69 U.S. healthcare organizations in the TriNetX electronic health record.

**Participants:**

Adults aged ≥50 years with ≥1 outpatient visit and sufficient clinical/laboratory data. Individuals with prior diagnoses of cognitive impairment, cerebrovascular disease, advanced liver disease, malignancy, schizophrenia, or substance use disorders were excluded. Two matched cohorts were constructed: one with 3,546,833 individuals with MRFs and 3,546,833 healthy controls, and another with 525,844 individuals with MASLD/MASH and 525,844 with MRFs only. Matching was based on age, sex, race, and ethnicity.

**Primary and secondary outcome measures:**

Incident MCI, VD, and AD, identified using ICD-10 codes, assessed at 5–20-year intervals. Odds ratios (ORs) with 95% confidence intervals (CIs) were calculated using logistic regression. Outcomes were prespecified.

**Results:**

Among 7,818,146 participants (mean [SD] age, 64.9 [8.8] years; 52.0% female), individuals with MRFs had higher odds of VD (OR, 1.65; 95% CI, 1.63–1.67), MCI (OR, 1.45; 95% CI, 1.42–1.48), and AD (OR, 1.21; 95% CI, 1.19–1.24) vs healthy controls. Compared to the MRF group, individuals with MASLD/MASH had lower odds of VD (OR, 0.86; 95% CI, 0.83–0.89) and AD (OR, 0.83; 95% CI, 0.78–0.88), but higher odds of MCI (OR, 1.37; 95% CI, 1.30–1.44); all *p* < .001.

**Conclusions:**

In this large, propensity-matched retrospective cohort study, MRFs were independently associated with significantly increased long-term odds of MCI, VD, and AD. MASLD/MASH demonstrated a divergent cognitive risk profile relative to MRFs alone—characterized by higher odds of MCI but paradoxically lower odds of VD and AD, a pattern that warrants cautious interpretation given potential competing mortality risk, survivor bias, and residual confounding. These findings suggest that MASLD/MASH is associated with a distinct cognitive trajectory, highlighting the importance of early cognitive surveillance in this population.

## Introduction

Metabolic dysfunction is a global health challenge with far-reaching consequences beyond its well-established association with cardiovascular disease and diabetes [1,2]. Emerging evidence suggests that MASLD, formerly non-alcoholic fatty liver disease (NAFLD), and its advanced form MASH may have systemic effects that extend to cognitive health [3–5]. The intersection of MRFs, liver disease, and cognitive outcomes may have significant implications, especially as the global prevalence of MASLD/MASH is increasing. Over the past three decades (1990–2019), the global prevalence of MASLD has grown substantially from 17.6% to 23.4% [6]. The global prevalence of MASLD among adults is currently estimated to be approximately 30%, while MASH affects about 5% of the adult population, with the highest prevalence observed in Latin America, MENA regions and South Asia [7,8].

The brain-liver axis has garnered increasing attention as a potential pathway linking metabolic dysfunction and neurocognitive decline [9,10]. MASLD and MASH are characterized by systemic inflammation, insulin resistance, and altered lipid metabolism [10,11]. These factors have been implicated in neurodegenerative processes [12,13]. However, epidemiologic data of neurocognitive impairment in individuals with MASLD/MASH remain limited. Observational studies have attempted to explore the association between MRFs, MASLD/MASH, and neurocognitive outcomes such as all-cause dementia, MCI and VD [14–18]. However, findings across studies are inconsistent with some reporting no association while others indicating either increased or decreased risk [14–18]. Furthermore, data on the long-term impact of MASLD/MASH remain limited, with prior studies often constrained by small sample sizes, heterogeneous populations, and a lack of long-term longitudinal follow-up.

To address these gaps, the primary objective of this study was to examine the independent associations between MRFs, MASLD/MASH, and the long-term incidence of MCI, VD, and AD in a large, propensity-matched retrospective cohort utilizing data from the TriNetX US Collaborative Network.

## Methods

### Data source

We conducted a retrospective cohort study using the TriNetX Analytics Network Platform (Cambridge, USA), federated research network that provides access to deidentified electronic health record data from over 118 million patients across 69 healthcare organizations (HCOs) in the United States. This database includes comprehensive demographic, clinical, diagnostic, and medication information. Diagnoses are recorded using the International Classification of Diseases, Ninth and Tenth Revisions (ICD-9 and ICD-10). The TriNetX platform includes built-in tools for cohort creation, outcome comparisons, and statistical analysis. The study followed STROBE guidelines for reporting observational studies.

### Inclusion and exclusion criteria

Adults aged ≥50 years at the index encounter were selected to enrich the study population for individuals at meaningful risk of incident cognitive decline and dementia, consistent with prior TriNetX-based and epidemiologic studies of neurodegenerative outcomes. Participants were included if they had ≥ 1 documented outpatient or office visit and sufficient clinical and laboratory data to determine exposure classification. Patients were excluded if they had pre-existing diagnoses of AD, VD, MCI, alcoholic liver disease, chronic viral hepatitis, or toxic liver disease. Additional exclusion criteria included cerebrovascular disease, heart failure, disseminated malignant neoplasms, schizophrenia or other psychotic disorders, or substance use disorders.

MRFs were defined as the presence of one or more of the following: fasting glucose ≥100 mg/dL (documented at any point prior to or at the index encounter), HDL cholesterol ≤40 mg/dL, triglyceride levels ≥150 mg/dL, essential (primary) hypertension, overweight or obesity, or a diagnosis of type 2 diabetes mellitus. MRFs were analyzed as a composite exposure; individual component-level analyses were not performed, as the TriNetX platform does not support granular stratification by individual metabolic risk factor within the propensity-matched framework.

**Fig 1:**
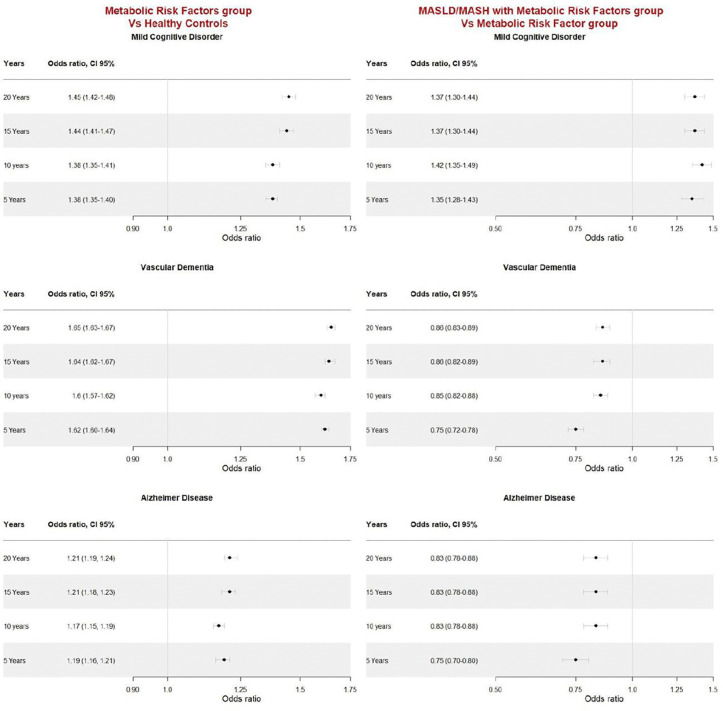
Flowchart of original and matched cohort creation from the TriNetX Network Platform.

MASLD and MASH were identified using ICD-10 codes K76.0 and K75.81, respectively (Supplementary Table 17 in S1 File). As TriNetX relies on administrative coding from electronic health records, the diagnosis of MASLD/MASH reflects clinical documentation by treating providers and may have been established through laboratory evaluation, imaging, or histological assessment; however, the specific diagnostic modality (e.g., liver biopsy, imaging, laboratory-based algorithms) cannot be ascertained from the available data. Information on fibrosis stage or histological disease severity was not available within the TriNetX platform. Detailed definitions and diagnostic codes for inclusion, exclusion, and exposure classification are provided in Fig 1 and Supplementary Table 17 in S1 File.

### Exposure classification and study group

To evaluate the association between metabolic status, MASLD/MASH and cognitive outcomes, participants were stratified into three mutually exclusive groups based on the presence of MRFs and a diagnosis of MASLD or MASH:

(1) Healthy Controls (HC): No history of MRFs, MASLD, or MASH.
(2) MRFs-only: One or more MRFs without a diagnosis of MASLD or MASH.
(3) MASLD-MRFs: Diagnosed with MASLD or MASH and at least one MRF.

### Study outcomes and follow-up timeframe

The primary outcomes of interest were VD, MCI, and AD, identified using ICD-10 codes. The index date was defined as the first day a participant met inclusion criteria. The analysis window began one day after the index event, and patients whose index event occurred 20 or more years before the analysis were excluded. Outcomes were tracked over varying follow-up durations, categorized as follows: (1) up to 5 years (1–1,825 days), (2) up to 10 years (1–3,650 days), (3) up to 15 years (1–5,475 days), and (4) up to 20 years (1–7,300 days).

### Statistical analysis

All statistical analyses were performed using TriNetX Live. Two sets of comparisons were made. Baseline characteristics of patients in the comparison groups were summarized as means with standard deviations (SD), medians with interquartile ranges (IQR), or proportions, as appropriate. Propensity score matching (1:1) was performed using greedy nearest neighbor methods based on eight variables: age at index, current age, sex, race (White, Black, Asian), and ethnicity (Hispanic or Latino). Balance was assessed using standardized differences (<0.1 threshold). Logistic regression was used to calculate odds ratios (ORs) with 95% confidence intervals (CIs), as the TriNetX platform does not currently support Cox proportional hazards or competing risk regression models. We acknowledge that logistic regression does not account for time-to-event or differential censoring, and that ORs may overestimate effect size when outcome prevalence is not rare. Given the low absolute event rates observed (all < 2%), the degree of overestimation is expected to be modest. Patients were censored at death or last available data point. A 2-sided *P* < 0.05 was considered statistically significant.

### Ethical approval

The TriNetX platform complies with HIPAA and U.S. federal regulations. As such, studies conducted through TriNetX do not require institutional review board approval or informed consent.

## Results

### Cohort structure and baseline characteristics

Following the application of inclusion and exclusion criteria, three mutually exclusive study groups were defined: (1) the Healthy Control Group (HC; *n* = 7,228,315), (2) the Metabolic Risk Factor Group (MRFs-only; *n* = 3,648,323), and (3) the MASLD/MASH with Metabolic Risk Factors Group (MASLD-MRFs; *n* = 525,354). After 1:1 propensity score matching (PSM) across relevant follow-up intervals, cohorts were successfully balanced with respect to age, sex, race, and ethnicity.

Baseline demographic characteristics were comparable across all matched cohorts. In the comparison between the HC and MRFs-only groups, the mean age was approximately 64 years, with males comprising approximately 46% of each cohort. The majority of participants in both groups were White (~63%) and identified as not Hispanic or Latino (~60%). In the comparison between the MASLD-MRFs and MRFs-only groups, the mean age at index event was approximately 60 years, with males accounting for approximately 40% of each cohort. Most participants were White (~70%) and not Hispanic or Latino (~58%). Summary demographic data are presented in Tables 1 and 2, with full baseline characteristics available in the Supplementary Materials in S1 File.

**Table 1:** Demographics of Metabolic Risk Factors vs. Healthy Controls after propensity score matching at 20 years.

Groups	After propensity score matching				
	Demographics	Mean ± SD	Patients (%)	*p*-Value	SD
HC	Current Age	70.4 + /- 10.4	3,427,277 (100)	<0.001	0.111
MRFs		69.3 + /- 9.6	3,427,277 (100)		
HC	Age at Index	64.7 + /- 10.3	3,427,277 (100)	<0.001	0.135
MRFs		63.4 + /- 9.1	3,427,277 (100)		
HC	Male		1,539,394 (44.9)	<0.001	0.018
MRFs			1,509,001 (44.0)		
HC	Female		1,756,016 (51.2)	<0.001	0.025
MRFs			1,798,422 (52.5)		
HC	Hispanic or Latino		257,664 (7.5)	<0.001	0.039
MRFs			293,933 (8.6)		
HC	White		2,243,931 (65.5)	<0.001	0.060
MRFs			2,145,583 (62.6)		
HC	African American		340,196 (9.9)	<0.001	0.128
MRFs			482,545 (14.1)		
HC	Asian		124,889 (3.6)	<0.001	0.026
MRFs			141,885 (4.1)		
HC	Other race		103,148 (3.0)	<0.001	0.073
MRFs			150,079 (4.4)		

Abbreviations: HC, healthy controls; MRFs, metabolic risk factors.

**Table 2:** Demographics of MASLD/MASH with Metabolic Risk Factors vs. Metabolic Risk Factors only after propensity score matching at 20 years.

Groups	After propensity score matching				
	Demographics	Mean ± SD	Patients % of Cohort	*p*-Value	SD
MRFs	Current Age	66.4 + /- 8.9	534,592 (100)	<0.001	0.089
MASLD/MASH-MRFs		65.6 + /- 9.4	534,592 (100)		
MRFs	Age at Index	61.8 + /- 8.5	534,592 (100)	<0.001	0.133
MASLD/MASH-MRFs		60.6 + /- 9.5	534,592 (100)		
MRFs	Male		192,871 (36.1)	<0.001	0.038
MASLD/MASH-MRFs			202,694 (37.9)		
MRFs	Female		312,879 (58.5)	<0.001	0.017
MASLD/MASH-MRFs			308,427 (57.7)		
MRFs	Hispanic or Latino		297,137 (55.6)	<0.001	0.126
MASLD/MASH-MRFs			330,517 (61.8)		
MRFs	White		357,587 (66.9)	<0.001	0.067
MASLD/MASH-MRFs			374,313 (70.0)		
MRFs	African American		35,634 (6.7)	<0.001	0.037
MASLD/MASH-MRFs			40,766 (7.6)		
MRFs	Asian		30,753 (5.8)	<0.001	0.043
MASLD/MASH-MRFs			25,602 (4.8)		
MRFs	Other race		31,350 (5.9)	<0.001	0.077
MASLD/MASH-MRFs			22,319 (4.2)		

Abbreviations: MRFs, metabolic risk factors; metabolic dysfunction–associated steatotic liver disease, MASLD; MASH, metabolic dysfunction–associated steatohepatitis.

### Comparison between MRFs-only and healthy control (HC) groups

Matched analysis revealed that individuals with MRFs had consistently higher odds of developing cognitive disorders compared to healthy controls across all follow-up durations. Within 5 years, the MRFs-only group demonstrated a 62% increased risk of VD (OR, 1.62, 95% CI, 1.60–1.64), a 38% increased risk of MCI (OR, 1.38, 95% CI, 1.35–1.40), and a 19% increased risk of AD (OR, 1.19, 95% CI: 1.16–1.21).

**Fig 2:**
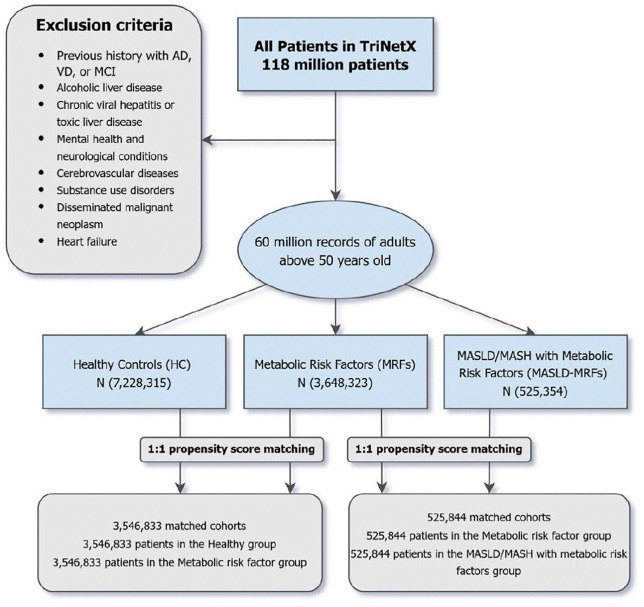
Odds Ratios with 95% Confidence Intervals for Cognitive and Dementia Outcomes by Metabolic Risk Factors and MASLD/MASH Over Time. Forest plots display point estimates (squares) and 95% CIs (horizontal lines) for the association between MRFs vs. healthy controls (upper panels) and MASLD/MASH-MRFs vs. MRFs alone (lower panels) for MCI, VD, and AD at 5, 10, 15, and 20 years of follow-up. The dashed vertical line at OR = 1.0 represents the null.

These associations remained stable over time. At 10 years, the odds of developing VD, MCI, and AD were 1.60 (95% CI, 1.57–1.62), 1.38 (95% CI, 1.35–1.41), and 1.17 (95% CI, 1.15–1.19), respectively. By 15 years, the odds increased to 1.64 for VD (95% CI, 1.62–1.67), 1.44 for MCI (95% CI, 1.41–1.47), and 1.21 for AD (95% CI, 1.18–1.23). At 20 years, the pattern remained consistent, with ORs of 1.65 for VD, 1.45 for MCI, and 1.21 for AD (all p < 0.001). These results are summarized in Table 3 and illustrated in Fig 2.

**Table 3:** Association of outcomes after propensity score matching.

Metabolic Risk Factors (MRFs) vs. Healthy Controls (HC)									
	Mild Cognitive Impairment			Vascular Dementia			Alzheimer Disease		
Follow-up	HC N (%)	MRFs N (%)	OR [95% CI]	HC N (%)	MRFs N (%)	OR [95% CI]	HC N (%)	MRFs N (%)	OR [95% CI]
**5-year**	17,336 (0.49)	23,785 (0.67)	1.38 (1.35, 1.40)	37,519 (1.06)	60,248 (1.70)	1.62 (1.60, 1.64)	18,489 (0.52)	21,907 (0.62)	1.19 (1.16, 1.21)
**10-year**	16,523 (0.47)	22,729 (0.65)	1.38 (1.35, 1.41)	36,268 (1.04)	57,499 (1.65)	1.60 (1.57, 1.62)	18,051 (0.52)	21,102 (0.61)	1.17 (1.15, 1.19)
**15-year**	16,539 (0.48)	23,692 (0.69)	1.44 (1.41, 1.47)	36,235 (1.06)	59,126 (1.73)	1.64 (1.62, 1.67)	18,062 (0.53)	21,764 (0.64)	1.21 (1.18, 1.23)
**20-year**	16,588 (0.48)	23,947 (0.70)	1.45 (1.42, 1.48)	36,313 (1.06)	59,522 (1.74)	1.65 (1.63, 1.67)	18,098 (0.53)	21,927 (0.64)	1.21 (1.19, 1.24)
**MASLD/MASH with MRFs (MASLD-MRFs) vs. Metabolic Risk Factors (MRFs)**									
	**Mild Cognitive Impairment**			**Vascular Dementia**			**Alzheimer Disease**		
**Follow-up**	**MRFs N (%)**	**MASLD-MRFs N (%)**	**OR [95% CI]**	**MRFs N (%)**	**MASLD-MRFs N (%)**	**OR [95% CI]**	**MRFs N (%)**	**MASLD-MRFs N (%)**	**OR [95% CI]**
**5-year**	2,080 (0.40)	2,808 (0.53)	1.35 (1.28, 1.43)	5,451 (1.04)	4,096 (0.78)	0.75 (0.72, 0.78)	1,840 (0.35)	1,379 (0.26)	0.75 (0.70, 0.80)
**10-year**	2,646 (0.49)	3,744 (0.69)	1.42 (1.35, 1.49)	6,079 (1.12)	5,183 (0.96)	0.85 (0.82, 0.88)	2,169 (0.40)	1,803 (0.33)	0.83 (0.78, 0.88)
**15-year**	2,815 (0.53)	3,839 (0.72)	1.37 (1.30, 1.44)	6,208 (1.16)	5,318 (1.00)	0.86 (0.82, 0.89)	2,254 (0.42)	1,868 (0.35)	0.83 (0.78, 0.88)
**20-year**	2,825 (0.53)	3,858 (0.72)	1.37 (1.30, 1.44)	6,219 (1.16)	5,335 (1.00)	0.86 (0.83, 0.89)	2,259 (0.42)	1,878 (0.35)	0.83 (0.78, 0.88)

*All p < 0.001. OR = odds ratio; CI = confidence interval; HC = healthy controls; MRFs = metabolic risk factors; MASLD = metabolic dysfunction–associated steatotic liver disease; MASH = metabolic dysfunction–associated steatohepatitis. N (%) = number of events and event rate within matched cohort.*

### Comparison between MASLD-MRFs and MRFs-only groups

Compared with individuals with MRFs alone, those with MASLD/MASH exhibited a distinct cognitive risk profile. Within 5 years of follow-up, the MASLD-MRFs group had a 25% lower risk of both VD (OR, 0.75; 95% CI, 0.72–0.78) and AD (OR, 0.75; 95% CI, 0.70–0.80), but a 35% increased risk of MCI (OR, 1.35; 95% CI, 1.28–1.43).

At 10 years, the odds of VD and AD remained reduced (ORs, 0.85 and 0.83, respectively), while the risk of MCI remained elevated (OR, 1.42; 95% CI, 1.35–1.49). This pattern persisted through 15 and 20 years of follow-up, with risks of VD and AD reduced by 14–17% and MCI risk elevated by 37% (OR, 1.37; 95% CI, 1.30–1.44 for both intervals).

Full results are presented in Table 3 and visualized in Fig 2.

## Discussion

This study found that individuals with MRFs had significantly higher odds of developing VD, MCI, and AD compared with healthy controls (HC). In contrast, participants with MASLD/MASH demonstrated consistently lower odds of VD and AD, but higher odds of MCI, relative to those with MRFs alone. These associations remained stable across all follow-up durations. However, the lower odds of VD and AD in the MASLD/MASH group should be interpreted with caution, as several alternative explanations may account for this finding. Competing mortality risk (i.e., higher cardiovascular or liver-related mortality among MASLD/MASH patients before dementia onset), detection bias arising from more frequent healthcare encounters in MASLD/MASH patients, survivor bias, and residual confounding from unmeasured variables (e.g., medication exposure, disease severity) may all contribute to the observed pattern. We therefore recommend caution in interpreting the lower observed odds ratios that require further investigation with competing risk models and more granular covariate adjustment.

Prior research has identified a potential association between MASLD/MASH and cognitive decline; however, findings specific to all-cause dementia and AD remained limited and inconsistent. While some studies suggest an increased risk of dementia among individuals with MASLD/MASH, others report no association–or even a reduced risk–particularly for VD. In a prospective analysis of UK Biobank data with 13 years of follow-up, Bao *et al.* (2024) reported that MASLD was associated with an elevated risk of VD but not with AD or all-cause dementia. Notably, a subgroup analysis found that individuals with metabolic dysfunction–associated alcohol-related liver disease (MetALD)– a MASLD subtype involving alcohol intake above recommended limits–had a lower risk of AD compared with those without hepatic steatosis. Similarly, a recent meta-analysis involving over 890,000 individuals from six countries found that MASLD was associated with higher odds of MCI but lower odds of VD, with no significant association observed for AD or all-cause dementia.

Xiao *et al.* (2022) found that MASLD was associated with a reduced risk of dementia within the first five years of follow-up, a finding that may reflect interactions between the conditions through interventions such as weight loss. Alternatively, the early stages of subclinical dementia are often characterized by unintentional weight loss, which can lead to regression of MASLD and complicate the detection of associations between MASLD and incident dementia, potentially resulting in reverse causation. This bidirectional interplay underscores the complexity of the relationship between liver pathology and cognitive outcomes. Collectively, current evidence–including the results of our study–suggests that MASLD and its subtypes, influenced with dynamic metabolic and lifestyle factors, may exert differential effects on cognitive trajectories. Further mechanistic research is warranted to delineate these relationships more precisely.

Experimental models of MASLD and NASH in rodents offer mechanistic insight into the observed associations between liver disease and cognitive impairment. Rodent studies have demonstrated that hepatic injury induced by high-fat, high-cholesterol, or high-sugar diets triggers pronounced neuroinflammatory changes. These alterations are accompanied by impairments in synaptic plasticity, reduced metabolic and neurotransmitter activity within the hippocampus and prefrontal cortex, and behavioral deficits in learning, memory, and mood regulation. Several liver-derived mediators–including lipocalin-2, hyperammonemia, and gut microbiota dysbiosis–have been identified as potential drivers of neuroinflammation and neurodegeneration along the liver-brain axis. These preclinical findings recapitulate key cognitive and pathological features of human MASLD/NASH and support biologically plausible pathways linking metabolic liver disease to cognitive decline [19–23].

Compared with healthy controls, individuals with MRFs exhibited significantly elevated risks of VD, MCI, and AD across all follow-up intervals. Although metabolic syndrome (MetS) has been implicated in the pathogenesis of dementia, the literature remains inconclusive. A 2019 meta-analysis of six longitudinal studies found no significant association between MetS and dementia [24]. More recent studies, however, have demonstrated increased dementia risk in individuals with MetS. For instance, Fan *et al.* found that increased dementia incidence was observed primarily among individuals with worsening MetS, while those with persistent MetS appeared to adapt to its physiological impact over time [25]. Several studies also suggest a cumulative effect, in which the number of metabolic abnormalities correlates positively with dementia risk [26,27]. Associations specific to AD are inconsistent–some studies indicate a reduced odds, others no association [25,28], and still some suggest an increased risk [29]. Cognitive impairment has also been linked to individual MetS components, including obesity, diabetes, and hypertension. Nonetheless, the current body of literature is constrained by varying definitions of MetS, heterogeneity in cognitive assessments, and potential survival bias [30]. Further research with standardized definitions and precise phenotyping is needed to clarify these relationships.

This study has several limitations that merit careful consideration. As a retrospective cohort study relying on administrative data, it is subject to inherent risks of selection bias and residual confounding. Propensity score matching was restricted to demographic variables (age, sex, race, and ethnicity) available within the TriNetX platform. Important potential confounders, including education level, socioeconomic status, smoking status, physical activity, alcohol intake, body mass index, baseline cognitive function, APOE genotype, and medication exposures (e.g., statins, GLP-1 receptor agonists, antihypertensives, and antidiabetics), were not available for inclusion in the matching algorithm. The absence of these covariates represents a significant source of residual confounding. Individuals with MASLD/MASH may receive more frequent medical surveillance and earlier cardiovascular risk management, which could contribute to the observed differences in cognitive outcomes. The dataset was derived from the TriNetX Research Network, which includes 69 U.S.-based healthcare organizations. As such, the findings may not be generalizable to non-U.S. populations or settings with differing healthcare access, socioeconomic conditions, or population health profiles. Additionally, the use of electronic health records (EHRs) and administrative coding (ICD-10) introduces the risk of exposure and outcome misclassification, as clinical diagnoses may not always be accurately recorded. While propensity score matching was employed to balance cohorts on demographic factors (age, sex, race, and ethnicity), unmeasured confounders—such as genetic predisposition (e.g., Apolipoprotein E genotype), lifestyle behaviors (e.g., physical activity, alcohol intake, smoking), and disease severity or duration—could have influenced observed associations.

The use of logistic regression rather than time-to-event models (e.g., Cox proportional hazards or Fine-Gray competing risk regression) is a notable limitation, as it does not account for differential censoring or competing mortality. Competing risk bias is particularly relevant to the interpretation of the paradoxically lower odds of VD and AD in the MASLD/MASH group: individuals with MASLD/MASH may experience higher cardiovascular or liver-related mortality before developing dementia, thereby attenuating the observed association with late-onset cognitive outcomes. Cause-specific mortality data were not available to stratify by competing causes of death. Additionally, our exposure classification was based on a single time-point assessment. Without time-varying exposure modeling, metabolic risk factors developing during follow-up would be missed, and transient MRFs would be treated equivalently to chronic ones. This study also lacked longitudinal data on MASLD/MASH management and did not capture dynamic changes in metabolic risk factor profiles or weight-related regression of hepatic steatosis, factors that may significantly affect cognitive trajectories. Moreover, the cognitive outcome definitions were based solely on ICD-10 diagnostic codes without clinical adjudication or neuropsychological evaluation. ICD-based ascertainment may miss early or mild cases, misclassify dementia subtypes, and is subject to inter-institutional variability in coding practices, which is particularly relevant for MCI. Despite these limitations, the study’s large sample size, robust matching methodology, and extended follow-up periods strengthen the validity and consistency of the observed associations.

In summary, this study highlights a complex relationship between metabolic dysfunction, hepatic steatosis, and cognitive decline. While MRFs were associated with increased risks of VD, MCI, and AD, MASLD/MASH exhibited a divergent profile—reduced odds of VD and AD but elevated odds of MCI. These findings underscore the need for nuanced, individualized risk stratification accounting for competing risks and suggest that hepatic and metabolic factors may be important, and currently underrecognized, contributors to dementia risk. Prospective studies with detailed phenotyping and mechanistic investigation are needed to fully clarify these associations.

## Conclusion

This study highlights the complex interplay between metabolic risk factors, MASLD/MASH, and long-term cognitive outcomes. With respect to MCI, individuals with MRFs had a 38–45% higher odds compared with healthy controls, and those with MASLD/MASH had a 35–42% higher odds of MCI compared with MRFs alone. This finding supports emerging evidence that cognitive impairments may develop early in the course of metabolic liver disease, often before the onset of advanced fibrosis or cirrhosis, and can affect domains such as memory, executive function, attention, and psychomotor speed. With respect to dementia outcomes (VD and AD), MRFs were associated with 17–65% higher odds compared with healthy controls. In contrast, MASLD/MASH was associated with lower observed odds of both VD and AD relative to MRFs alone; however, this finding should be interpreted with caution, as competing mortality risk, survivor bias, and residual confounding may account for the observed differences. A deeper understanding of the mechanisms underlying these associations is critical for improving risk stratification and guiding patient counseling.

The underlying mechanisms are likely multifactorial, encompassing systemic neuroinflammation, insulin resistance, vascular dysfunction, disruption of the gut-liver-brain axis, and structural or functional abnormalities of the hippocampus. The differential cognitive risk profiles observed between metabolic risk factors and MASLD/MASH further emphasize the need for individualized approaches to the management of both metabolic and neurocognitive health.

These findings support the role of early cognitive screening in individuals with MASLD/MASH, particularly in those with coexisting metabolic comorbidities or progressive liver disease. Future research should focus on clarifying these pathways and addressing key knowledge gaps regarding the contribution of metabolic dysfunction to neurocognitive decline.

## Supporting information

S1 File Supplementary demographic and outcome data. Includes demographic characteristics comparing MRFs vs. HC and MASLD/MASH-MRFs vs. MRFs before and after propensity score matching across 5, 10, 15, and 20 years of follow-up; outcome associations for MCI, VD, and AD before propensity score matching; and ICD-10 diagnostic codes used in this study (Supplement Tables 1–17).
(DOCX)

S2 File STROBE checklist. Completed STROBE checklist for reporting of observational cohort studies.
(DOCX)
