# Supplementary material for: Metabolic risk and metabolic dysfunction–associated steatotic liver disease and steatohepatitis in cognitive decline: A retrospective cohort study
Source: PLoS One. 2026 Jul 9;21(7):e0353160. doi: 10.1371/journal.pone.0353160 (PMC13349133; doi:10.1371/journal.pone.0353160)
Supplement: S1 File — Includes demographic characteristics comparing MRFs vs. HC and MASLD/MASH-MRFs vs. MRFs before and after propensity score matching across 5, 10, 15, and 20 years of follow-up; outcome associations for MCI, VD, and AD before propensity score matching; and ICD-10 diagnostic codes used in this study (Supplement Tables 1–17). (DOCX) [file pone.0353160.s001.docx]

**Table of Contents**

Supplement Table 1-4: Demographic characteristics comparing Metabolic Risk Factors (MRFs) vs. Healthy Controls (HC) before propensity score matching at 5, 10, 15, and 20 years follow-up.

Supplement Table 5: Association of outcomes (Mild Cognitive Impairment, Vascular Dementia, Alzheimer's) comparing MRFs vs. HC before propensity score matching across 5, 10, 15, and 20 years.

Supplement Table 6-9: Demographic characteristics comparing MASLD/MASH (MASLD-MRFs) vs. Metabolic Risk Factors (MRFs) before propensity score matching at 5, 10, 15, and 20 years follow-up.

Supplement Table 10: Association of outcomes (Mild Cognitive Impairment, Vascular Dementia, Alzheimer's) comparing MASLD-MRFs vs. MRFs before propensity score matching across 5, 10, 15, and 20 years.

Supplement Table 11-13: Demographic characteristics comparing Metabolic Risk Factors (MRFs) vs. Healthy Controls (HC) after propensity score matching at 5, 10, and 15 years follow-up.

Supplement Table 14-16: Demographic characteristics comparing MASLD/MASH (MASLD-MRFs) vs. Metabolic Risk Factors (MRFs) after propensity score matching at 5, 10, and 15 years follow-up.

Supplement Table 17: ICD-10 Codes for various diseases and conditions.

**Supplement Table 1. Demographic characteristics when comparing before propensity score matching: Metabolic risk factors (MRFs) vs Healthy controls (HC) at 5 years follow up**

| **Groups** | **Before propensity score matching** | | | | | |
| --- | --- | --- | --- | --- | --- | --- |
|  | **Demographics** | **Mean ± SD** | **Patients (%)** | **p-Value** | **Std**  **diff.** |  |
| HC | Current Age | 66.3 +/- 11.3 | 7,228,315 (100) | <0.001 | 0.315 |  |
| MRFs |  | 69.6 +/- 9.6 | 3,648,323 (100) |  |  |  |
| HC | Age at Index | 58.9 +/- 12.1 | 7,228,315 (100) | <0.001 | 0.442 |  |
| MRFs |  | 63.7 +/- 9.2 | 3,648,323 (100) |  |  |  |
| HC | Male |  | 2,893,837 (41.39) | <0.001 | 0.092 |  |
| MRFs |  |  | 1,608,085 (46.1) |  |  |  |
| HC | Female |  | 4,098,422 (58.61) | <0.001 | 0.092 |  |
| MRFs |  |  | 1,879,246 (53.9) |  |  |  |
| HC | Hispanic or Latino |  | 439,254 (6.08) | <0.001 | 0.109 |  |
| MRFs |  |  | 322,289 (8.9) |  |  |  |
| HC | Not Hispanic or Latino |  | 4,266,450 (59.02) | <0.001 | <0.001 |  |
| MRFs |  |  | 2,128,592 (59.0) |  |  |  |
| HC | Unknown ethnicity |  | 2,522,611 (34.9) | <0.001 | 0.06 |  |
| MRFs |  |  | 1,156,927 (32.1) |  |  |  |
| HC | White |  | 4,655,000 (64.4) | <0.001 | 0.052 |  |
| MRFs |  |  | 2,233,605 (61.9) |  |  |  |
| HC | American Indian or Alaska native |  | 14,080 (0.19) | <0.001 | 0.03 |  |
| MRFs |  |  | 12,544 (0.35) |  |  |  |
| HC | Black or African American |  | 467,116 (6.46) | <0.001 | 0.273 |  |
| MRFs |  |  | 533,997 (14.8) |  |  |  |
| HC | Native Hawaiian or Other Pacific Islander |  | 12,689 (0.18) | <0.001 | 0.068 |  |
| MRFs |  |  | 21,544 (0.59) |  |  |  |
| HC | Asian |  | 220,685 (3.05) | <0.001 | 0.032 |  |
| MRFs |  |  | 131,207 (3.64) |  |  |  |
| HC | Other race |  | 274,080 (3.79) | <0.001 | 0.019 |  |
| MRFs |  |  | 150,111 (4.2) |  |  |  |
| HC | Unknown race |  | 1,584,665 (21.92) | <0.001 | 0.192 |  |
| MRFs |  |  | 524,792 (14.6) |  |  |  |

**Supplement Table 2. Demographic characteristics when comparing before propensity score matching: Metabolic risk factors (MRFs) vs Healthy controls (HC) at 10 years follow up**

| **Groups** | **Before propensity score matching** | | | | |
| --- | --- | --- | --- | --- | --- |
|  | **Demographics** | **Mean ± SD** | **Patients (%)** | **p-Value** | **Std**  **diff.** |
| HC | Current Age | 66.4 +/- 11.3 | 6,687,239 (100) | <0.001 | 0.289 |
| MRFs |  | 69.5 +/- 9.6 | 3,606,708 (100) |  |  |
| HC | Age at Index | 59.0 +/- 12.2 | 6,687,239 (100) | <0.001 | 0.426 |
| MRFs |  | 63.6 +/- 9.1 | 3,606,708 (100) |  |  |
| HC | Male |  | 2,679,635 (40.1) | <0.001 | 0.087 |
| MRFs |  |  | 1,599,971 (44.4) |  |  |
| HC | Female |  | 3,771,061 (56.4) | <0.001 | 0.082 |
| MRFs |  |  | 1,886,323 (52.3) |  |  |
| HC | Hispanic or Latino |  | 420,273 (6.3) | <0.001 | 0.097 |
| MRFs |  |  | 318,774 (8.8) |  |  |
| HC | Not Hispanic or Latino |  | 4,130,197 (61.8) | <0.001 | 0.015 |
| MRFs |  |  | 2,254,263 (62.5) |  |  |
| HC | Unknown ethnicity |  | 2,136,769 (32.0) | <0.001 | 0.072 |
| MRFs |  |  | 1,033,671 (28.7) |  |  |
| HC | White |  | 4,253,930 (63.6) | <0.001 | 0.045 |
| MRFs |  |  | 2,215,702 (61.4) |  |  |
| HC | American Indian or Alaska native |  | 13,854 (0.2) | <0.001 | 0.014 |
| MRFs |  |  | 9,967 (0.3) |  |  |
| HC | Black or African American |  | 457,608 (6.8) | <0.001 | 0.286 |
| MRFs |  |  | 570,354 (15.8) |  |  |
| HC | Native Hawaiian or Other Pacific Islander |  | 11,857 (0.2) | <0.001 | 0.082 |
| MRFs |  |  | 26,363 (0.7) |  |  |
| HC | Asian |  | 201,805 (3.0) | <0.001 | 0.063 |
| MRFs |  |  | 151,184 (4.2) |  |  |
| HC | Other race |  | 276,639 (4.1) | <0.001 | 0.014 |
| MRFs |  |  | 159,220 (4.4) |  |  |
| HC | Unknown race |  | 1,471,546 (22.0) | <0.001 | 0.235 |
| MRFs |  |  | 473,918 (13.1) |  |  |

**Supplement Table 3. Demographic characteristics when comparing before propensity score matching: Metabolic risk factors (MRFs) vs Healthy controls (HC) at 15 years follow up**

| **Groups** | **Before propensity score matching** | | | | |
| --- | --- | --- | --- | --- | --- |
|  | **Demographics** | **Mean ± SD** | **Patients (%)** | **p-Value** | **Std**  **diff.** |
| HC | Current Age | 66.4 +/- 11.3 | 6,458,323 (100) | <0.001 | 0.287 |
| MRFs |  | 69.5 +/- 9.6 | 3,558,311 (100) |  |  |
| HC | Age at Index | 59.0 +/- 12.2 | 6,458,323 (100) | <0.001 | 0.425 |
| MRFs |  | 63.6 +/- 9.2 | 3,558,311 (100) |  |  |
| HC | Male |  | 2,579,529 (39.9) | <0.001 | 0.088 |
| MRFs |  |  | 1,575,580 (44.3) |  |  |
| HC | Female |  | 3,642,272 (56.4) | <0.001 | 0.082 |
| MRFs |  |  | 1,862,319 (52.3) |  |  |
| HC | Hispanic or Latino |  | 403,661 (8.8) | <0.001 | 0.097 |
| MRFs |  |  | 313,428 (6.3) |  |  |
| HC | Not Hispanic or Latino |  | 3,953,360 (62.3) | <0.001 | 0.022 |
| MRFs |  |  | 2,216,047 (61.2) |  |  |
| HC | Unknown ethnicity |  | 2,101,302 (32.5) | <0.001 | 0.079 |
| MRFs |  |  | 1,028,836 (28.9) |  |  |
| HC | White |  | 4,069,457 (63.0) | <0.001 | 0.037 |
| MRFs |  |  | 2,178,725 (61.2) |  |  |
| HC | American Indian or Alaska native |  | 12,508 (0.2) | <0.001 | 0.014 |
| MRFs |  |  | 9,222 (0.3) |  |  |
| HC | Black or African American |  | 455,624 (7.1) | <0.001 | 0.283 |
| MRFs |  |  | 569,574 (16.0) |  |  |
| HC | Native Hawaiian or Other Pacific Islander |  | 11,262 (0.2) | <0.001 | 0.082 |
| MRFs |  |  | 25,675 (0.7) |  |  |
| HC | Asian |  | 197,091 (3.1) | <0.001 | 0.062 |
| MRFs |  |  | 149,940 (4.2) |  |  |
| HC | Other race |  | 247,947 (3.8) | <0.001 | 0.022 |
| MRFs |  |  | 151,755 (4.3) |  |  |
| HC | Unknown race |  | 1,464,434 (22.7) | <0.001 | 0.246 |
| MRFs |  |  | 473,420 (13.3) |  |  |

**Supplement Table 4. Demographic characteristics when comparing before propensity score matching: Metabolic risk factors (MRFs) vs Healthy controls (HC) at 20 years follow up**

| **Groups** | **Before propensity score matching** | | | | |
| --- | --- | --- | --- | --- | --- |
|  | **Demographics** | **Mean ± SD** | **Patients (%)** | **p-Value** | **Std**  **diff.** |
| HC | Current Age | 66.4 +/- 11.3 | 6,458,323 (100) | <0.001 | 0.287 |
| MRFs |  | 69.5 +/- 9.6 | 3,558,311 (100) |  |  |
| HC | Age at Index | 59.0 +/- 12.2 | 6,458,323 (100) | <0.001 | 0.425 |
| MRFs |  | 63.6 +/- 9.2 | 3,558,311 (100) |  |  |
| HC | Male |  | 2,579,529 (39.9) | <0.001 | 0.088 |
| MRFs |  |  | 1,575,580 (44.3) |  |  |
| HC | Female |  | 3,642,272 (56.4) | <0.001 | 0.082 |
| MRFs |  |  | 1,862,319 (52.3) |  |  |
| HC | Hispanic or Latino |  | 403,661 (6.3) | <0.001 | 0.097 |
| MRFs |  |  | 313,428 (8.8) |  |  |
| HC | Not Hispanic or Latino |  | 3,953,360 (61.2) | <0.001 | 0.022 |
| MRFs |  |  | 2,216,047 (62.3) |  |  |
| HC | Unknown ethnicity |  | 2,101,302 (32.5) | <0.001 | 0.079 |
| MRFs |  |  | 1,028,836 (28.9) |  |  |
| HC | White |  | 4,069,457 (61.2) | <0.001 | 0.037 |
| MRFs |  |  | 2,178,725 (63.0) |  |  |
| HC | American Indian or Alaska native |  | 12,508 (0.3) | <0.001 | 0.014 |
| MRFs |  |  | 9,222 (0.2) |  |  |
| HC | Black or African American |  | 455,624 (7.1) | <0.001 | 0.283 |
| MRFs |  |  | 569,574 (16.0) |  |  |
| HC | Native Hawaiian or Other Pacific Islander |  | 11,262 (0.2) | <0.001 | 0.082 |
| MRFs |  |  | 25,675 (0.7) |  |  |
| HC | Asian |  | 197,091 (3.1) | <0.001 | 0.062 |
| MRFs |  |  | 149,940 (4.2) |  |  |
| HC | Other race |  | 247,947 (3.8) | <0.001 | 0.022 |
| MRFs |  |  | 151,755 (4.3) |  |  |
| HC | Unknown race |  | 1,464,434 (22.7) | <0.001 | 0.246 |
| MRFs |  |  | 473,420 (13.3) |  |  |

**Supplement Table 5. Association of outcomes among metabolic risk group (MRFs) compared to healthy controls (HC) before PSM.**

|  | **Mild Cognitive Impairment** | | | | **Vascular dementia** | | | | **Alzheimer** | | | |
| --- | --- | --- | --- | --- | --- | --- | --- | --- | --- | --- | --- | --- |
|  | HC  N (%) | MRFs N (%) | OR [95%CI] | p-Value | HC  N (%) | MRFs N (%) | OR [95%CI] | p-Value | HC  N (%) | MRFs N (%) | OR [95%CI] | p-Value |
| **5- year** | 27752 (0.38) | 24637 (0.68) | 1.76 (1.73, 1.80) | <0.001 | 54727 (0.76) | 62774 (1.72) | 2.30 (2.27, 2.32) | <0.001 | 27667 (0.38) | 23062 (0.63) | 1.66 (1.63, 1.69) | <0.001 |
| **10-year** | 24891 (0.37) | 23663 (0.65) | 1.77 (1.74, 1.80) | <0.001 | 48998 (0.73) | 61485 (1.7) | 2.35 (2.32, 2.38) | <0.001 | 25084 (0.38) | 22545 (0.63) | 1.67 (1.64, 1.70) | <0.001 |
| **15- year** | 24664 (0.38) | 24627 (0.69) | 1.82 (1.79, 1.85) | <0.001 | 48672 (0.75) | 63378 (1.78) | 2.39 (2.36, 2.42) | <0.001 | 24857 (0.38) | 23426 (0.66) | 1.72 (1.69, 1.75) | <0.001 |
| **20-year** | 26162 (0.39) | 25490 (0.71) | 1.81 (1.78, 1.85) | <0.001 | 50803 (0.76) | 64528 (1.79) | 2.38 (2.35, 2.41) | <0.001 | 25893 (0.39) | 23840 (0.66) | 1.71 (1.68, 1.74) | <0.001 |

**Supplement Table 6. Demographic characteristics when comparing before propensity score matching: MASLD/MASH (MASLD-MRFs) vs Metabolic risk factors (MRFs) at 5 years follow up.**

| **Groups** | **Before propensity score matching** | | | | |
| --- | --- | --- | --- | --- | --- |
|  | **Demographics** | **Mean ± SD** | **Patients (%)** | **p-Value** | **Std**  **diff.** |
| MRFs | Current Age | 69.5 +/- 9.6 | 3,595,374 (100) | <0.001 | 0.419 |
| MASLD-MRFs |  | 65.5 +/- 9.4 | 525,354 (100) |  |  |
| MRFs | Age at Index | 62.8 +/- 9.1 | 3,595,374 (100) | <0.001 | 0.243 |
| MASLD-MRFs |  | 60.5 +/- 9.5 | 525,354 (100) |  |  |
| MRFs | Male |  | 1,601,026 (46.1) | <0.001 | 0.132 |
| MASLD-MRFs |  |  | 199,754 (39.8) |  |  |
| MRFs | Female |  | 1,873,899 (53.9) | <0.001 | 0.109 |
| MASLD-MRFs |  |  | 302,150 (60.2) |  |  |
| MRFs | Hispanic or Latino |  | 323,275 (9.0) | <0.001 | 0.161 |
| MASLD-MRFs |  |  | 74,244 (14.1) |  |  |
| MRFs | Not Hispanic or Latino |  | 2,123,737 (59.0) | <0.001 | 0.026 |
| MASLD-MRFs |  |  | 2,123,737 (57.8) |  |  |
| MRFs | Unknown ethnicity |  | 1,148,362 (32.0) | <0.001 | 0.085 |
| MASLD-MRFs |  |  | 147,400 (28.1) |  |  |
| MRFs | White |  | 2,222,254 (61.8) | <0.001 | 0.193 |
| MASLD-MRFs |  |  | 372,492 (70.9) |  |  |
| MRFs | American Indian or Alaska native |  | 12,511 (0.35) | <0.001 | 0.003 |
| MASLD-MRFs |  |  | 1,907 (0.36) |  |  |
| MRFs | Black or African American |  | 544,795 (15.1) | <0.001 | 0.267 |
| MASLD-MRFs |  |  | 36,132 (6.9) |  |  |
| MRFs | Native Hawaiian or Other Pacific Islander |  | 16,116 (0.45) | <0.001 | 0.028 |
| MASLD-MRFs |  |  | 3,444 (0.45) |  |  |
| MRFs | Asian |  | 123,350 (3.4) | <0.001 | 0.013 |
| MASLD-MRFs |  |  | 19,308 (3.7) |  |  |
| MRFs | Other race |  | 152,771 (4.3) | <0.001 | 0.005 |
| MASLD-MRFs |  |  | 22,818 (4.3) |  |  |
| MRFs | Unknown race |  | 523,577 (14.6) | <0.001 | 0.040 |
| MASLD-MRFs |  |  | 69,253 (13.2) |  |  |

**Supplement Table 7. Demographic characteristics when comparing before propensity score matching: MASLD/MASH (MASLD-MRFs) vs Metabolic risk factors (MRFs) at 10 years follow up.**

| **Groups** | **Before propensity score matching** | | | | |
| --- | --- | --- | --- | --- | --- |
|  | **Demographics** | **Mean ± SD** | **Patients (%)** | **p-Value** | **Std**  **diff.** |
| MRFs | Current Age | 69.5 +/- 9.6 | 3,606,705 (100) | <0.001 | 0.410 |
| MASLD-MRFs |  | 65.6 +/- 9.4 | 540,987 (100) |  |  |
| MRFs | Age at Index | 63.6 +/- 9.1 | 3,606,705 (100) | <0.001 | 0.325 |
| MASLD-MRFs |  | 60.6 +/- 9.5 | 540,987 (100) |  |  |
| MRFs | Male |  | 1,599,971 (44.4) | <0.001 | 0.130 |
| MASLD-MRFs |  |  | 205,419 (38.0) |  |  |
| MRFs | Female |  | 1,886,320 (52.3) | <0.001 | 0.109 |
| MASLD-MRFs |  |  | 312,099 (57.7) |  |  |
| MRFs | Hispanic or Latino |  | 318,774 (8.8) | <0.001 | 0.156 |
| MASLD-MRFs |  |  | 74,359 (13.7) |  |  |
| MRFs | Not Hispanic or Latino |  | 2,254,26 (62.5) | <0.001 | 0.011 |
| MASLD-MRFs |  |  | 335,341 (62.0) |  |  |
| MRFs | Unknown ethnicity |  | 1,033,669 (28.7) | <0.001 | 0.100 |
| MASLD-MRFs |  |  | 131,287 (24.3) |  |  |
| MRFs | White |  | 2,215,699 (70.1) | <0.001 | 0.183 |
| MASLD-MRFs |  |  | 379,064 (61.4) |  |  |
| MRFs | American Indian or Alaska native |  | 9,967 (0.3) | <0.001 | 0.005 |
| MASLD-MRFs |  |  | 1,645 (0.3) |  |  |
| MRFs | Black or African American |  | 570,354 (15.8) | <0.001 | 0.260 |
| MASLD-MRFs |  |  | 40,784 (7.5) |  |  |
| MRFs | Native Hawaiian or Other Pacific Islander |  | 26,363 (0.7) | <0.001 | 0.014 |
| MASLD-MRFs |  |  | 4,645 (0.9) |  |  |
| MRFs | Asian |  | 151,184 (4.2) | <0.001 | 0.028 |
| MASLD-MRFs |  |  | 25,770 (4.8) |  |  |
| MRFs | Other race |  | 159,220 (4.4) | 0.100 | 0.002 |
| MASLD-MRFs |  |  | 23,616 (4.4) |  |  |
| MRFs | Unknown race |  | 473,918 (13.1) | <0.001 | 0.031 |
| MASLD-MRFs |  |  | 65,463 (12.1) |  |  |

**Supplement Table 8. Demographic characteristics when comparing before propensity score matching: MASLD/MASH (MASLD-MRFs) vs Metabolic risk factors (MRFs) at 15 years follow up.**

| **Groups** | **Before propensity score matching** | | | | |
| --- | --- | --- | --- | --- | --- |
|  | **Demographics** | **Mean ± SD** | **Patients (%)** | **p-Value** | **Std**  **diff.** |
| MRFs | Current Age | 69.5 +/- 9.6 | 3,558,539 (100) | <0.001 | 0.409 |
| MASLD-MRFs |  | 65.6 +/- 9.4 | 534,593 (100) |  |  |
| MRFs | Age at Index | 63.6 +/- 9.2 | 3,558,539 (100) | <0.001 | 0.323 |
| MASLD-MRFs |  | 60.6 +/- 9.5 | 534,593 (100) |  |  |
| MRFs | Male |  | 1,575,857 (44.3) | <0.001 | 0.130 |
| MASLD-MRFs |  |  | 202,694 (37.9) |  |  |
| MRFs | Female |  | 1,862,270 (52.3) | <0.001 | 0.108 |
| MASLD-MRFs |  |  | 308,428 (57.7) |  |  |
| MRFs | Hispanic or Latino |  | 313,475 (8.8) | <0.001 | 0.156 |
| MASLD-MRFs |  |  | 73,330 (13.7) |  |  |
| MRFs | Not Hispanic or Latino |  | 2,216,188 (62.3) | <0.001 | 0.009 |
| MASLD-MRFs |  |  | 330,517 (61.8) |  |  |
| MRFs | Unknown ethnicity |  | 1,028,876 (28.9) | <0.001 | 0.101 |
| MASLD-MRFs |  |  | 130,746 (24.5) |  |  |
| MRFs | White |  | 2,178,857 (61.2) | <0.001 | 0.186 |
| MASLD-MRFs |  |  | 374,313 (70.0) |  |  |
| MRFs | American Indian or Alaska native |  | 9,228 (0.3) | <0.001 | 0.005 |
| MASLD-MRFs |  |  | 1,539 (0.3) |  |  |
| MRFs | Black or African American |  | 569,554 (16.0) | <0.001 | 0.262 |
| MASLD-MRFs |  |  | 40,766 (7.6) |  |  |
| MRFs | Native Hawaiian or Other Pacific Islander |  | 25,675 (0.7) | <0.001 | 0.015 |
| MASLD-MRFs |  |  | 4,588 (0.9) |  |  |
| MRFs | Asian |  | 149,968 (4.2) | <0.001 | 0.028 |
| MASLD-MRFs |  |  | 25,603 (4.8) |  |  |
| MRFs | Other race |  | 151,748 (4.3) | 0.003 | 0.004 |
| MASLD-MRFs |  |  | 22,319 (4.2) |  |  |
| MRFs | Unknown race |  | 473,509 (13.3) | <0.001 | 0.032 |
| MASLD-MRFs |  |  | 65,465 (12.2) |  |  |

**Supplement Table 9. Demographic characteristics when comparing before propensity score matching: MASLD/MASH (MASLD-MRFs) vs Metabolic risk factors (MRFs) at 20 years follow up.**

| **Groups** | **Before propensity score matching** | | | | |
| --- | --- | --- | --- | --- | --- |
|  | **Demographics** | **Mean ± SD** | **Patients (%)** | **p-Value** | **Std**  **diff.** |
| MRFs | Current Age | 69.5 +/- 9.6 | 3,558,539 (100) | <0.001 | 0.409 |
| MASLD-MRFs |  | 65.6 +/- 9.4 | 534,593 (100) |  |  |
| MRFs | Age at Index | 63.6 +/- 9.2 | 3,558,539 (100) | <0.001 | 0.323 |
| MASLD-MRFs |  | 60.6 +/- 9.5 | 534,593 (100) |  |  |
| MRFs | Male |  | 1,575,857 (44.3) | <0.001 | 0.130 |
| MASLD-MRFs |  |  | 202,694 (37.9) |  |  |
| MRFs | Female |  | 1,862,270 (52.3) | <0.001 | 0.108 |
| MASLD-MRFs |  |  | 308,428 (57.7) |  |  |
| MRFs | Hispanic or Latino |  | 313,475 (8.8) | <0.001 | 0.156 |
| MASLD-MRFs |  |  | 73,330 (13.7) |  |  |
| MRFs | Not Hispanic or Latino |  | 2,216,188 (62.3) | <0.001 | 0.009 |
| MASLD-MRFs |  |  | 330,517 (61.8) |  |  |
| MRFs | Unknown ethnicity |  | 1,028,876 (28.9) | <0.001 | 0.101 |
| MASLD-MRFs |  |  | 130,746 (24.5) |  |  |
| MRFs | White |  | 2,178,857 (61.2) | <0.001 | 0.186 |
| MASLD-MRFs |  |  | 374,313 (70.0) |  |  |
| MRFs | American Indian or Alaska native |  | 9,228 (0.3) | <0.001 | 0.005 |
| MASLD-MRFs |  |  | 1,539 (0.3) |  |  |
| MRFs | Black or African American |  | 569,554 (16.0) | <0.001 | 0.262 |
| MASLD-MRFs |  |  | 40,766 (7.6) |  |  |
| MRFs | Native Hawaiian or Other Pacific Islander |  | 25,675 (0.7) | <0.001 | 0.015 |
| MASLD-MRFs |  |  | 4,588 (0.9) |  |  |
| MRFs | Asian |  | 149,968 (4.2) | <0.001 | 0.028 |
| MASLD-MRFs |  |  | 25,603 (4.8) |  |  |
| MRFs | Other race |  | 151,748 (4.3) | 0.003 | 0.004 |
| MASLD-MRFs |  |  | 22,319 (4.2) |  |  |
| MRFs | Unknown race |  | 473,509 (13.3) | <0.001 | 0.032 |
| MASLD-MRFs |  |  | 65,465 (12.2) |  |  |

**Supplement Table 10. Association of outcomes among MASLD/MASH (MASLD-MRFs) with Metabolic risk factors (MRFs) before PSM.**

|  | **Mild Cognitive Impairment** | | | | **Vascular dementia** | | | | | **Alzheimer** | | | | |
| --- | --- | --- | --- | --- | --- | --- | --- | --- | --- | --- | --- | --- | --- | --- |
|  | MRFs  N (%) | MASLD-MRFs  N (%) | OR [95%CI] | p-Value | MRFs  N (%) | MASLD-MRFs  N (%) | OR [95%CI] | p-Value | MRFs  N (%) | | MASLD-MRFs  N (%) | OR [95%CI] | p-Value |  |
| **5- year** | 16545 (0.46) | 2764 (0.53) | 1.14 (1.10, 1.19) | <0.001 | 48645 (1.35) | 4068 (0.77) | 0.57 (0.55, 0.59) | <0.001 | 17284 (0.48) | | 1355 (0.26) | 0.54 (0.51, 0.57) | <0.001 |  |
| **10-year** | 23663 (0.66) | 3744 (0.69) | 1.06 (1.02, 1.09) | <0.001 | 61485 (1.7) | 5183 (0.96) | 0.56 (0.54, 0.58) | <0.001 | 22545 (0.63) | | 1803 (0.33) | 0.53 (0.51, 0.56) | <0.001 |  |
| **15- year** | 24630 (0.38) | 3839 (0.69) | 1.04 (1.00, 1.07) | 0.052 | 63393 (0.75) | 5318 (1.78) | 0.55 (0.54, 0.57) | <0.001 | 23435 (0.38) | | 1868 (0.66) | 0.53 (0.51, 0.56) | <0.001 |  |
| **20-year** | 24885 (0.70) | 3858 (0.72) | 1.03 (1.00, 1.07) | 0.051 | 63794 (1.79) | 5335 (1.00) | 0.54 (0.54, 0.57) | <0.001 | 23599 (0.66) | | 1878 (0.35) | 0.53 (0.50, 0.55) | <0.001 |  |

**Supplement Table 11. Demographic characteristics when comparing after propensity score matching: Metabolic risk factors (MRFs) vs Healthy controls (HC) at 5 years follow up**

| **Groups** | **After propensity score matching** | | | | |
| --- | --- | --- | --- | --- | --- |
|  | **Demographics** | **Mean ± SD** | **Patients (%)** | **p-Value** | **Std**  **diff.** |
| HC | Current Age | 70.5 +/- 10.3 | 3,546,833 (100) | <0.001 | 0.098 |
| MRFs |  | 69.5 +/- 9.6 | 3,546,833 (100) |  |  |
| HC | Age at Index | 64.7 +/- 10.2 | 3,546,833 (100) | <0.001 | 0.120 |
| MRFs |  | 63.5 +/- 9.1 | 3,546,833 (100) |  |  |
| HC | Male |  | 1,629,026 (47.7) | <0.001 | 0.03 |
| MRFs |  |  | 1,576,338 (46.0) |  |  |
| HC | Female |  | 1,886,323 (52.3) | <0.001 | 0.04 |
| MRFs |  |  | 1,850,243 (54.0) |  |  |
| HC | Hispanic or Latino |  | 280,950 (7.9) | <0.001 | 0.032 |
| MRFs |  |  | 312,249 (8.8) |  |  |
| HC | Not Hispanic or Latino |  | 2,164,217 (61.0) | <0.001 | 0.042 |
| MRFs |  |  | 2,091,783 (59.0) |  |  |
| HC | Unknown ethnicity |  | 1,101,666 (31.1) | <0.001 | 0.025 |
| MRFs |  |  | 1,142,801 (32.2) |  |  |
| HC | White |  | 2,328,009 (65.6) | <0.001 | 0.062 |
| MRFs |  |  | 2,222,805 (62.7) |  |  |
| HC | American Indian or Alaska native |  | 5,283 (0.15) | <0.001 | 0.041 |
| MRFs |  |  | 12,531 (0.35) |  |  |
| HC | Black or African American |  | 334,734 (9.4) | <0.001 | 0.137 |
| MRFs |  |  | 486,734 (13.7) |  |  |
| HC | Native Hawaiian or Other Pacific Islander |  | 4,762 (0.13) | <0.001 | 0.078 |
| MRFs |  |  | 21,536 (0.6) |  |  |
| HC | Asian |  | 124,553 (3.5) | <0.001 | 0.008 |
| MRFs |  |  | 129,643 (3.7) |  |  |
| HC | Other race |  | 114,258 (3.2) | <0.001 | 0.052 |
| MRFs |  |  | 149,276 (4.2) |  |  |
| HC | Unknown race |  | 635,234 (18.0) | <0.001 | 0.08 |
| MRFs |  |  | 524,308 (14.8) |  |  |

**Supplement Table 12. Demographic characteristics when comparing after propensity score matching: Metabolic risk factors (MRFs) vs Healthy controls (HC) at 10 years follow up**

| **Groups** | **After propensity score matching** | | | | |
| --- | --- | --- | --- | --- | --- |
|  | **Demographics** | **Mean ± SD** | **Patients (%)** | **p-Value** | **Std**  **diff.** |
| HC | Current Age | 70.4 +/- 10.4 | 3,486,175 (100) | <0.001 | 0.109 |
| MRFs |  | 69.3 +/- 9.6 | 3,486,175 (100) |  |  |
| HC | Age at Index | 64.7 +/- 10.3 | 3,486,175 (100) | <0.001 | 0.134 |
| MRFs |  | 63.4 +/- 9.1 | 3,486,175 (100) |  |  |
| HC | Male |  | 1,576,200 (45.2) | <0.001 | 0.021 |
| MRFs |  |  | 1,539,114 (44.1) |  |  |
| HC | Female |  | 1,776,703 (51.0) | <0.001 | 0.029 |
| MRFs |  |  | 1,827,234 (52.4) |  |  |
| HC | Hispanic or Latino |  | 263,197 (7.5) | <0.001 | 0.041 |
| MRFs |  |  | 301,965 (8.7) |  |  |
| HC | Not Hispanic or Latino |  | 2,230,421 (64.0) | <0.001 | 0.031 |
| MRFs |  |  | 2,177,841(62.5) |  |  |
| HC | Unknown ethnicity |  | 992,557 (28.5) | <0.001 | 0.009 |
| MRFs |  |  | 1,006,369 (28.9) |  |  |
| HC | White |  | 2,298,370 (65.9) | <0.001 | 0.066 |
| MRFs |  |  | 2,188,224 (62.8) |  |  |
| HC | American Indian or Alaska native |  | 5,137 (0.1) | <0.001 | 0.030 |
| MRFs |  |  | 9,950 (0.3) |  |  |
| HC | Black or African American |  | 340,902 (9.8) | <0.001 | 0.130 |
| MRFs |  |  | 487,464 (14.0) |  |  |
| HC | Native Hawaiian or Other Pacific Islander |  | 4,419 (0.1) | <0.001 | 0.095 |
| MRFs |  |  | 26,332 (0.8) |  |  |
| HC | Asian |  | 125,606 (3.6) | <0.001 | 0.026 |
| MRFs |  |  | 143,359 (4.1) |  |  |
| HC | Other race |  | 114,141 (3.3) | <0.001 | 0.065 |
| MRFs |  |  | 157,853 (4.5) |  |  |
| HC | Unknown race |  | 597,600 (17.1) | <0.001 | 0.099 |
| MRFs |  |  | 472,993 (13.6) |  |  |

**Supplement Table 13. Demographic characteristics when comparing after propensity score matching: Metabolic risk factors (MRFs) vs Healthy controls (HC) at 15 years follow up**

| **Groups** | **After propensity score matching** | | | | |
| --- | --- | --- | --- | --- | --- |
|  | **Demographics** | **Mean ± SD** | **Patients (%)** | **p-Value** | **Std**  **diff.** |
| HC | Current Age | 70.4 +/- 10.4 | 3,427,277 (100) | <0.001 | 0.111 |
| MRFs |  | 69.3 +/- 9.6 | 3,427,277 (100) |  |  |
| HC | Age at Index | 64.7 +/- 10.3 | 3,427,277 (100) | <0.001 | 0.135 |
| MRFs |  | 63.4 +/- 9.1 | 3,427,277 (100) |  |  |
| HC | Male |  | 1,539,394 (44.9) | <0.001 | 0.018 |
| MRFs |  |  | 1,509,001 (44.0) |  |  |
| HC | Female |  | 1,756,016 (51.2) | <0.001 | 0.025 |
| MRFs |  |  | 1,798,422 (52.5) |  |  |
| HC | Hispanic or Latino |  | 257,664 (7.5) | <0.001 | 0.039 |
| MRFs |  |  | 293,933 (8.6) |  |  |
| HC | Not Hispanic or Latino |  | 2,180,050 (63.6) | <0.001 | 0.028 |
| MRFs |  |  | 2,133,487 (62.3) |  |  |
| HC | Unknown ethnicity |  | 989,563 (28.9) | <0.001 | 0.007 |
| MRFs |  |  | 999,857 (29.2) |  |  |
| HC | White |  | 2,243,931 (65.5) | <0.001 | 0.060 |
| MRFs |  |  | 2,145,583 (62.6) |  |  |
| HC | American Indian or Alaska native |  | 4,675 (0.1) | <0.001 | 0.029 |
| MRFs |  |  | 9,204 (0.3) |  |  |
| HC | Black or African American |  | 340,196 (9.9) | <0.001 | 0.128 |
| MRFs |  |  | 482,545 (14.1) |  |  |
| HC | Native Hawaiian or Other Pacific Islander |  | 4,306 (0.1) | <0.001 | 0.094 |
| MRFs |  |  | 25,637 (0.7) |  |  |
| HC | Asian |  | 124,889 (3.6) | <0.001 | 0.026 |
| MRFs |  |  | 141,885 (4.1) |  |  |
| HC | Other race |  | 103,148 (3.0) | <0.001 | 0.073 |
| MRFs |  |  | 150,079 (4.4) |  |  |
| HC | Unknown race |  | 606,132 (17.7) | <0.001 | 0.107 |
| MRFs |  |  | 472,344 (13.8) |  |  |

**Supplement Table 14. Demographic characteristics when comparing after propensity score matching: MASLD/MASH (MASLD-MRFs) vs Metabolic risk factors (MRFs) at 5 years follow up.**

| **Groups** | **After propensity score matching** | | | | |
| --- | --- | --- | --- | --- | --- |
|  | **Demographics** | **Mean ± SD** | **Patients (%)** | **p-Value** | **Std**  **diff.** |
| MRFs | Current Age | 66.2 +/- 9.0 | 525,844 (100) | <0.001 | 0.075 |
| MASLD-MRFs |  | 65.5 +/- 9.4 | 525,844 (100) |  |  |
| MRFs | Age at Index | 61.6 +/- 8.4 | 525,844 (100) | <0.001 | 0.123 |
| MASLD-MRFs |  | 60.5 +/- 9.5 | 525,844 (100) |  |  |
| MRFs | Male |  | 202,130 (40.6) | <0.001 | 0.009 |
| MASLD-MRFs |  |  | 199,754 (39.8) |  |  |
| MRFs | Female |  | 296,280 (59.4) | <0.001 | 0.022 |
| MASLD-MRFs |  |  | 302,130 (60.2) |  |  |
| MRFs | Hispanic or Latino |  | 78,124 (14.8) | <0.001 | 0.021 |
| MASLD-MRFs |  |  | 74,224 (14.1) |  |  |
| MRFs | Not Hispanic or Latino |  | 281,984 (53.7) | <0.001 | 0.083 |
| MASLD-MRFs |  |  | 303,710 (57.8) |  |  |
| MRFs | Unknown ethnicity |  | 165,226 (31.5) | <0.001 | 0.074 |
| MASLD-MRFs |  |  | 147,400 (28.1) |  |  |
| MRFs | White |  | 350,625 (66.7) | <0.001 | 0.09 |
| MASLD-MRFs |  |  | 372,472 (70.9) |  |  |
| MRFs | American Indian or Alaska native |  | 2,121 (0.40) | <0.001 | 0.007 |
| MASLD-MRFs |  |  | 1,907 (0.36) |  |  |
| MRFs | Black or African American |  | 32,068 (6.1) | <0.001 | 0.031 |
| MASLD-MRFs |  |  | 36,132 (6.9) |  |  |
| MRFs | Native Hawaiian or Other Pacific Islander |  | 2,645 (0.50) | <0.001 | 0.02 |
| MASLD-MRFs |  |  | 3,444 (0.66) |  |  |
| MRFs | Asian |  | 18,640 (3.6) | <0.001 | 0.007 |
| MASLD-MRFs |  |  | 19,308 (3.7) |  |  |
| MRFs | Other race |  | 30,339 (5.8) | <0.001 | 0.065 |
| MASLD-MRFs |  |  | 22,818 (4.3) |  |  |
| MRFs | Unknown race |  | 88,896 (16.9) | <0.001 | 0.105 |
| MASLD-MRFs |  |  | 69,253 (13.2) |  |  |

**Supplement Table 15. Demographic characteristics when comparing after propensity score matching: MASLD/MASH (MASLD-MRFs) vs Metabolic risk factors (MRFs) at 10 years follow up.**

| **Groups** | **After propensity score matching** | | | | |
| --- | --- | --- | --- | --- | --- |
|  | **Demographics** | **Mean ± SD** | **Patients (%)** | **p-Value** | **Std**  **diff.** |
| MRFs | Current Age | 66.2 +/- 9.0 | 540,986 (100) | <0.001 | 0.068 |
| MASLD-MRFs |  | 65.6 +/- 9.4 | 540,986 (100) |  |  |
| MRFs | Age at Index | 61.6 +/- 8.6 | 540,986 (100) | <0.001 | 0.111 |
| MASLD-MRFs |  | 60.6 +/- 9.5 | 540,986 (100) |  |  |
| MRFs | Male |  | 197,651 (36.5) | <0.001 | 0.030 |
| MASLD-MRFs |  |  | 205,419 (38.0) |  |  |
| MRFs | Female |  | 316,138 (58.4) | <0.001 | 0.015 |
| MASLD-MRFs |  |  | 312,098 (57.7) |  |  |
| MRFs | Hispanic or Latino |  | 92,681 (17.1) | <0.001 | 0.094 |
| MASLD-MRFs |  |  | 74,358 (13.7) |  |  |
| MRFs | Not Hispanic or Latino |  | 304,724 (56.3) | <0.001 | 0.115 |
| MASLD-MRFs |  |  | 335,341 (62.0) |  |  |
| MRFs | Unknown ethnicity |  | 143,581 (26.5) | <0.001 | 0.052 |
| MASLD-MRFs |  |  | 131,287 (24.3) |  |  |
| MRFs | White |  | 364,712 (67.4) | <0.001 | 0.057 |
| MASLD-MRFs |  |  | 379,064 (70.1) |  |  |
| MRFs | American Indian or Alaska native |  | 1,533 (0.3) | 0.047 | 0.004 |
| MASLD-MRFs |  |  | 1,645 (0.3) |  |  |
| MRFs | Black or African American |  | 35,805 (6.6) | <0.001 | 0.036 |
| MASLD-MRFs |  |  | 40,784 (7.5) |  |  |
| MRFs | Native Hawaiian or Other Pacific Islander |  | 3,853 (0.7) | <0.001 | 0.017 |
| MASLD-MRFs |  |  | 4,645 (0.9) |  |  |
| MRFs | Asian |  | 28,226 (5.2) | <0.001 | 0.021 |
| MASLD-MRFs |  |  | 25,769 (4.8) |  |  |
| MRFs | Other race |  | 30,861 (5.7) | <0.001 | 0.061 |
| MASLD-MRFs |  |  | 23,616 (4.4) |  |  |
| MRFs | Unknown race |  | 75,996 (14.0) | <0.001 | 0.058 |
| MASLD-MRFs |  |  | 65,463 (12.1) |  |  |

**Supplement Table 16. Demographic characteristics when comparing after propensity score matching: MASLD/MASH (MASLD-MRFs) vs Metabolic risk factors (MRFs) at 15 years follow up.**

| **Groups** | **After propensity score matching** | | | | |
| --- | --- | --- | --- | --- | --- |
|  | **Demographics** | **Mean ± SD** | **Patients (%)** | **p-Value** | **Std**  **diff.** |
| MRFs | Current Age | 66.4 +/- 8.9 | 534,592 (100) | <0.001 | 0.089 |
| MASLD-MRFs |  | 65.6 +/- 9.4 | 534,592 (100) |  |  |
| MRFs | Age at Index | 61.8 +/- 8.5 | 534,592 (100) | <0.001 | 0.133 |
| MASLD-MRFs |  | 60.6 +/- 9.5 | 534,592 (100) |  |  |
| MRFs | Male |  | 192,871 (36.1) | <0.001 | 0.038 |
| MASLD-MRFs |  |  | 202,694 (37.9) |  |  |
| MRFs | Female |  | 312,879 (58.5) | <0.001 | 0.017 |
| MASLD-MRFs |  |  | 308,427 (57.7) |  |  |
| MRFs | Hispanic or Latino |  | 297,137 (55.6) | <0.001 | 0.127 |
| MASLD-MRFs |  |  | 330,517 (61.8) |  |  |
| MRFs | Not Hispanic or Latino |  | 297,137 (55.6) | <0.001 | 0.127 |
| MASLD-MRFs |  |  | 330,517 (61.8) |  |  |
| MRFs | Unknown ethnicity |  | 139,534 (26.1) | <0.001 | 0.038 |
| MASLD-MRFs |  |  | 130,746 (24.5) |  |  |
| MRFs | White |  | 357,587 (66.9) | <0.001 | 0.067 |
| MASLD-MRFs |  |  | 374,313 (70.0) |  |  |
| MRFs | American Indian or Alaska native |  | 1,378 (0.3) | 0.003 | 0.006 |
| MASLD-MRFs |  |  | 1,539 (0.3) |  |  |
| MRFs | Black or African American |  | 35,634 (6.7) | <0.001 | 0.037 |
| MASLD-MRFs |  |  | 40,766 (7.6) |  |  |
| MRFs | Native Hawaiian or Other Pacific Islander |  | 3,388 (0.6) | <0.001 | 0.026 |
| MASLD-MRFs |  |  | 4,588 (0.9) |  |  |
| MRFs | Asian |  | 30,753 (5.8) | <0.001 | 0.043 |
| MASLD-MRFs |  |  | 25,602 (4.8) |  |  |
| MRFs | Other race |  | 31,350 (5.9) | <0.001 | 0.077 |
| MASLD-MRFs |  |  | 22,319 (4.2) |  |  |
| MRFs | Unknown race |  | 74,502 (13.9) | <0.001 | 0.050 |
| MASLD-MRFs |  |  | 65,465 (12.2) |  |  |

**Supplement Table 17. ICD-10 Codes for different disorders.**

| **Disease** | **ICD10 Codes** |
| --- | --- |
| Vascular Dementia | **(UMLS: ICD10CM: F01),** |
| Mild Cognitive Disorder | **(UMLS: ICD10CM: G31.84)** |
| Alzheimer's Disease | **(UMLS: ICD10CM: G30)** |
| Metabolic-Associated Steatotic Liver Disease (MASLD) | **(UMLS: ICD10CM: K76.0)** |
| Metabolic Dysfunction-Associated Steatohepatitis (MASH) | **(UMLS: ICD10CM: K75.81)** |
| Alcoholic Liver Disease | **(UMLS: ICD10CM: K70.0)** |
| Chronic Viral Hepatitis | **(UMLS: ICD10CM: B18.9)** |
| Toxic Liver Disease | **(UMLS: ICD10CM: K71)** |
| Cerebrovascular Disease | **(UMLS: ICD10CM: I67.9)** |
| Heart Failure | **(UMLS: ICD10CM: I50.9)** |
| Disseminated Malignant Neoplasm | **(UMLS: ICD10CM: C80.0)** |
| Schizophrenia | **(UMLS: ICD10CM: F20)** |
| Substance Use Disorders | **(UMLS: ICD10CM: F19.20)** |
| Primary Hypertension | **(UMLS: ICD10CM: I10)** |
| Overweight | **(UMLS: ICD10CM: E66.3)** |
| Obesity | **(UMLS: ICD10CM: E66.9)** |
| Type 2 Diabetes Mellitus | **(UMLS: ICD10CM: E11)** |
